# Daylight Saving Time Transitions and Road Traffic Accidents

**DOI:** 10.1155/2010/657167

**Published:** 2010-06-27

**Authors:** Tuuli Lahti, Esa Nysten, Jari Haukka, Pekka Sulander, Timo Partonen

**Affiliations:** ^1^Department of Mental Health and Substance Abuse Services, National Institute for Health and Welfare, 00271 Helsinki, Finland; ^2^Federation of Accident Insurance Institutions, Helsinki, Finland

## Abstract

Circadian rhythm disruptions may have harmful impacts on health. Circadian rhythm disruptions caused by jet lag compromise the quality and amount of sleep and may lead to a variety of symptoms such as fatigue, headache, and loss of attention and alertness. Even a minor change in time schedule may cause considerable stress for the body. Transitions into and out of daylight saving time alter the social and environmental timing twice a year. According to earlier studies, this change in time-schedule leads to sleep disruption and fragmentation of the circadian rhythm. Since sleep deprivation decreases motivation, attention, and alertness, transitions into and out of daylight saving time may increase the amount of accidents during the following days after the transition. We studied the amount of road traffic accidents one week before and one week after transitions into and out of daylight saving time during years from 1981 to 2006. Our results demonstrated that transitions into and out of daylight saving time did not increase the number of traffic road accidents.

## 1. Introduction

Daylight saving time (DST) is used to better match the activity peaks of a population with the daylight hours. The original purpose of DST was energy conservation. However, recent research indicates that DST does not substantially reduce energy consumption and may even increase it. The research has also shown that transitions into and out of DST cause minor jet lag symptoms such as sleep disruption, fragmentation of the circadian rhythm, and fatigue [1–3]. Since DST does not serve its original purpose and also causes a variety of disruptive symptoms, it is debatable whether there is any reason to maintain the practice.

Transitions into and out of DST change our social timing quickly, but affect the timing of body changes more slowly. According to our earlier results, it takes several days to adjust to new time schedules resulting from DST transitions [1–3]. Since sleep deprivation and circadian rhythm disruptions are known to decrease attention and alertness, DST transitions may lead to higher accident rates. Only a few studies have explored the impact of DST transitions on accident rates [4–8], and the results are inconsistent. Some reports suggest that DST transitions increase accident rates, while others suggest that DST transitions decrease them [4–7]. Our study showed that transitions into and out of DST did not increase the number of accidents requiring hospitalization [8]. However, DST transitions may increase the number of less severe incidents which do not necessiate hospital treatment. Herein, our aim was to assess whether DST transitions increase the number of traffic accidents during the first week after transitions. We hypothesized that if sleep disruption caused by DST transitions increases accident rates, this increase will be seen during the first week after the transitions. This assumption is based on our earlier studies which indicated that DST transitions cause only minor and short-term symptoms: the healthy subjects recovered from the mild rhythm disruption caused by DST within one week.

## 2. Methods

We studied the number of accidents one week before and one week after DST transitions from 1981 to 2006. The data used in this paper was based on nation-wide register of Finnish Motor Insurers' Centre. Finnish Motor Insurers' Centre collects harm reports from both police and insurers' and thus the Finnish Motor Insurers' Centre's data accuracy is higher than any other sources of accident data in Finland. Finnish Motor Insurers' centre's data has information for each day separately, and also the causes of accidents are known. The methods for data collection and reporting have not been changed since 1973 and thus the data can be accurately compared from year to year. The number of accidents was analysed using a Poisson regression model with a log-link function which is the standard method for analysing frequency data [9]. The proportion of personal injuries in all traffic accidents was modeled with logistic regression. In both models, the year (from 1981 to 2006) was used as a continuous explanatory variable, and the season (spring or autumn) and period of time shift (before or after DST transitions) as categorical explanatory variables. We modeled the effect of year using a natural spline smoother. Splines are a good choice for smoothing because of the simplicity of their construction, ease of use, and accuracy of evaluation, as well as their capacity to approximate complex shapes through curve fitting [10]. The significance of the explanatory variables was tested with a likelihood ratio test. All modeling was carried out using R software ([11], www.r-project.org/).

## 3. Results

All interactions between explanatory variables were found to be significant when the number of traffic accidents was modeled (Table 1). Season did not essentially modify the very weak association between DST and number of accidents. However, both season and DST did modify how accident rates were associated with calendar year. For proportion of personal injuries we found two significant interactions: between season and DST, and season and year (Table 2).

## 4. Discussion

According to our results, transitions into and out of DST did not significantly increase the amount of traffic accidents. It now seems that DST transitions do not cause such detrimental consequences as accidents. The impact of DST transitions seems to be temporary and mild. However, those who are especially sensitive to circadian rhythm disruptions, such as patients suffering from seasonal affective disorder or bipolar disorder, may be more vulnerable to sudden changes in timing. In addition, those who already suffer from chronic sleep loss may suffer more from the additional sleep deprivation caused by DST transitions.

During the study period from 1981 to 2006, the proportion of personal injuries increased in spring but remained at the same level in autumn. According to our statistics, the amount of road traffic has increased from 1981 to 2006 and similarly the total number of traffic accidents. However, the number of personal injuries has decreased at the same time. This is probably the result of improved education and better traffic culture reducing the number of severe accidents.

## Figures and Tables

**Table 1:** Analysis of deviance table for number of traffic accidents. Poisson model with log-link function. Traffic intensity is included as background, and effect of year is modeled by using natural splines with *d* *f* = 4.

	*d* *f*	Estimate	Deviance	Residual *d* *f*	Residual Deviance	*P*(>|*χ*|)
Season	1	−0.12	29.71	102	5103.90	<.0001
DST	1	−0.17	19.99	101	5083.92	.8212
Year	4	0.26	1086.72	97	3997.20	<.0001
Road traffic total	1	−0.01	26.64	96	3960.56	<.0001

Interactions						
Season: DST	1	0.05	26.70	95	3933.86	<.0001
DST: Year	4	0.27	152.13	91	3781.73	<.0001
Season: Year	4	0.05	457.86	87	3323.87	<.0001

**Table 2:** Analysis of deviance table for proportion person injuries of all traffic accidents. Logistic regression model with logit link function. Traffic intensity is included as background, and effect of year is modeled by using natural splines with *d* *f* = 4.

	*d* *f*	Estimate	Deviance	Residual *d* *f*	Residual Deviance	*P*(>|*χ*|)
Season	1	0.60	369.73	102	426.95	<.0001
DST	1	0.05	0.01	101	426.94	.9347
Year	4	0.17	64.82	97	362.12	<.0001
Road traffic total	1	0.01	7.54	96	354.58	.0060

Interactions						
Season: DST	1	0.10	11.45	95	343.13	.0007
Season: Year	4	−0.43	87.45	91	255.68	<.0001
